# A Recombinant Acetylcholine Receptor α1 Subunit Extracellular Domain Is a Promising New Drug Candidate for Treatment Of Myasthenia Gravis

**DOI:** 10.3389/fimmu.2022.809106

**Published:** 2022-06-03

**Authors:** Konstantinos Lazaridis, Maria Fernandez-Santoscoy, Vasiliki Baltatzidou, Jan-Olof Andersson, Richard Christison, John Grünberg, Socrates Tzartos, Björn Löwenadler, Charlotte Fribert

**Affiliations:** ^1^ Department of Immunology, Hellenic Pasteur Institute, Athens, Greece; ^2^ Toleranzia AB, Göteborg, Sweden; ^3^ Department of Neurobiology, Hellenic Pasteur Institute, Athens, Greece; ^4^ Tzartos NeuroDiagnostics, Athens, Greece

**Keywords:** myasthenia gravis, acetylcholine receptor, autoimmune disease, antigen-specific immune tolerance, tolerogen, intravenous administration

## Abstract

**Background and Aims:**

Myasthenia gravis (MG) is a T-cell dependent antibody-mediated autoimmune disease in which the nicotinic acetylcholine receptor (AChR) is the major autoantigen, comprising several T and B cell auto-epitopes. We hypothesized that an efficacious drug candidate for antigen-specific therapy in MG should comprise a broad range of these auto-epitopes and be administered in a noninflammatory and tolerogenic context.

**Methods:**

We used a soluble mutated form of the extracellular domain of the α1 chain of the AChR (α1-ECD_m_), which represents the major portion of auto-epitopes involved in MG, and investigated, in a well-characterized rat model of experimental autoimmune myasthenia gravis (EAMG) whether its intravenous administration could safely and efficiently treat the autoimmune disease.

**Results:**

We demonstrated that intravenous administration of α1-ECD_m_ abrogates established EAMG, in a dose and time dependent manner, as assessed by clinical symptoms, body weight, and compound muscle action potential (CMAP) decrement. Importantly, the effect was more pronounced compared to drugs representing current standard of care for MG. The protein had a short plasma half-life, most of what could be recovered was sequestered in the liver, kidneys and spleen. Further, we did not observe any signs of toxicity or intolerability in animals treated with α1-ECD_m._

**Conclusion:**

We conclude that intravenous treatment with α1-ECD_m_ is safe and effective in suppressing EAMG. α1-ECD_m_ is in preclinical development as a promising new drug candidate for MG.

## 1 Introduction

Myasthenia gravis (MG) is a CD4^+^ T cell-dependent antibody-mediated autoimmune disease, which leads to destruction of the skeletal muscle nicotinic acetylcholine receptor (AChR) at the neuromuscular junction (1, 2) resulting in the hallmark MG symptoms of muscle weakness and fatigue. Antibodies against the AChR are found in a majority of patients (~85%), while fewer patients have antibodies against the muscle specific kinase (~9%), the low-density lipoprotein receptor-related protein 4 (~2%), or other less common targets (3). The AChR is a transmembrane glycoprotein composed of five subunits with a stoichiometry of (α1)_2_β1γδ in fetal or denervated muscles and (α1)_2_β1ϵδ in adult muscles. Each subunit has a highly structured extracellular domain (ECD), which contains the disease-relevant autoantibody binding sites. Among the different ECDs, that of the α1 subunit (α1-ECD) is the primary antibody target in the autoimmune attack (4–6) and most evidence so far suggests that the α1-ECD directed antibodies are the most pathogenic (7). Although MG is an antibody-mediated disease, high affinity autoantibody production by B cells is dependent on CD4+ T cell activity. Indeed, AChR-reactive CD4+ T cells have been found in MG patients, while T cell recognition of the AChR has been examined extensively and several studies have identified T-cell reactive auto-peptides, in particular from the α1-ECD (6–10). Taken together, both the antibody and T cell reactivities point to the α1-ECD as being a disease-specific antigen of particular interest in MG.

Currently, the most common therapeutic strategies for MG include the use of cholinesterase inhibitors, corticosteroids, mmunosuppressants, plasmapheresis, intravenous immunoglobulin, monoclonal antibodies or thymectomy (11, 12). These treatments are not disease-specific and can cause significant side-effects. They can alleviate symptoms, but they are not curative. Therefore, lifelong immunosuppressive therapy is often required but some patients may prove treatment refractory (13).

The ideal therapy would be disease-specific and target efficiently only the pathogenic autoreactive component of the immune system. Antigen-specific immune tolerization for treatment of autoimmune diseases may specifically abrogate autoimmunity without hampering normal immune function (14).

Experimental autoimmune MG (EAMG), which can be induced in rats by administration of AChR domains, represents a reliable animal model for the study of novel therapeutics against MG (15). Early attempts to induce antigen-specific immune tolerance in EAMG rats involved oral or nasal administration of AChR subunit domains (16–18). Both preventive, i.e. treatment before disease induction, and therapeutic treatment regimens have been explored, with the latter requiring higher antigen doses than the former to achieve a comparable effect (17). Mucosal administration of AChR-derived peptides, rather than whole protein domains, has also been tested with some positive results in EAMG mice (19, 20).

Induction of tolerance *via* intravenous injection (i.v. tolerance) is also possible. Notably, administration of soluble or nanoparticle-carried antigens *via* the intravenous route has recently rendered positive results against autoimmune diseases, such as multiple sclerosis, Graves’ disease, and celiac disease, in the clinic (21–23), pointing to the intravenous route of administration as promoting a tolerogenic setting suitable for antigen-specific immune tolerance approaches.

In this study, we sought to develop a soluble protein-based intravenous therapeutic approach for MG, suitable for translation to the clinic, combining the potential advantages of allowing patients’ own antigen processing and presentation.

To this end, we used a rat EAMG model induced with recombinant domains of the human AChR, previously used for the study of antigen-specific treatments (24, 25), and explored the therapeutic efficiency of intravenous antigen administration, which has not been studied in MG before. We found that intravenous administration had a robust therapeutic effect in EAMG, contrary to current MG therapeutics. We proceeded to characterize in detail several parameters affecting treatment efficacy, such as dose of antigen and frequency of administration. We conclude that this approach could be developed as a novel highly effective treatment for MG.

## 2 Materials and Methods

### 2.1 Experimental Animals

6- to 7-week-old female Lewis rats (weighing 120–135 g) were obtained from the animal breeding unit of the Department of Animal Models for Biomedical Research of the Hellenic Pasteur Institute. They were maintained in the rodent unit of the Department, in plastic cages with wire mesh lids and 4 cm thick wood-shavings bedding (four rats per 1,600 cm^2^ cage). Upon symptom manifestation they were provided with water gels and soft food at the bottom of the cages throughout the remaining experiment. All experiments described were approved by the Institute Ethics Board and conducted according to the regulations and guidelines for animal care (EU Directive 2010/63/EU for animal experiments).

### 2.2 Synthesis of α1-ECD_mt_ and α1-ECD_m_

α1-ECD_mt_ consists of the ECD of the human AChR α1 subunit, mutated by having its Cys-loop exchanged for that of the homologous acetylcholine binding protein from the snail *Lymnaea stagnalis*, and tagged with a Flag- and a 6-His-tag at its N- and C-terminal ends, respectively. α1-ECD_mt_ was expressed in the yeast *Pichia pastoris* as a soluble secreted polypeptide and purified by means of metal-affinity chromatography followed by size exclusion chromatography as previously described (26). Except for the Flag- and His-tags, the amino acid sequence of α1-ECD_m_ consists of the same elements as α1-ECD_mt_. α1-ECD_m_ was expressed in *Escherichia coli* strain NEB express (New England Biolabs Inc. USA) using a modified version of the pTrc99A-vector (Pharmacia AB, Sweden) harboring the km^r^ gene and in which the gene encoding α1-ECD_m_ was under transcriptional control by an IPTG-inducible Trp/Lac promoter. Briefly, *E. coli* cells were cultured in terrific broth (Thermofisher Scientific, USA) at 37°C and α1-ECD_m_ expression was induced with 1 mM IPTG at an OD_600_ of about 0.6. Following induction, α1-ECD_m_ accumulated in inclusion bodies in high quantities. α1-ECD_m_-containing inclusion bodies were purified by cell-disruption in lysis buffer (0.1 M Tris, 5 mM EDTA, pH 8.5) followed by repeated washings in 2 M Urea, 2% Triton X-100 in lysis buffer and finally solubilized in 40 mM Tris, 8 M Urea, 5 mM EDTA, pH 8.5. Refolding of α1-ECD_m_ was performed in 40 mM Tris, 50 mM NaCl, 1 M Urea, 10% Glycerol, 5% Sucrose pH 8.5 overnight at 4°C. Refolded α1-ECD_m_ was further purified by anion exchange chromatography on Q Sepharose FF at pH 7.4 and size exclusion chromatography (SEC) on Superdex 200 pg. Following the SEC step, α1-ECD_m_ purity was >90% with endotoxin levels below < 1 EU/mg. The overall yield of purified α1-ECD_m_ was about 80 mg/L of *E. coli* culture. Following sterile filtration, α1-ECD_m_ was frozen in storage buffer (30 mM NaP, 0.3 M NaCl, pH 7.4) and stored at -80°C.

### 2.3 Induction, Treatment, and Clinical Evaluation of EAMG

For induction of EAMG, rats were anaesthetized with 2% isoflurane supplemented with oxygen. They were injected subcutaneously in both hind footpads and at three sites in the lower back with a total of 80 μg α1-ECD_mt_ prepared as described in section 2.2, or PBS for controls, in CFA (Becton, Dickinson and Company) supplemented with 2 mg/ml inactivated Mycobacterium tuberculosis H37RA (Becton, Dickinson and Company), in a final volume of 250 μl. Serum samples were collected from the rats by tail vein blood sampling at different timepoints during the experiments and used for anti-AChR antibody quantitation as described in Section 2.7.

Regarding treatment administration, rats were treated intranasally or intravenously with α1-ECD_mt_ or α1-ECD_m_ starting 7, 21 or 40 days after EAMG induction. The amount of protein was 100 µg administered in a volume of 10 µl per nostril, or 100, 500 or 1,000 µg administered in a volume of 200 µl in tail vein. For the experiments comparing α1-ECD_mt_ with MG current standard of care, rats were treated with 1 mg methylprednisolone (Solumedrol, Pfizer) injected IP in a volume of 100 μl or 18.5 mg/Kg pyridostigmine (Mestinon, Meda Pharma GmbH), administered *via* oral gavage in a volume of 200 μl. These doses were higher than what is commonly used in clinical practice (27), but well tolerated in EAMG based on previous studies (28, 29). A list of all the applied regimens is shown in **Table 1**. Control animals received only PBS in all experiments.

**Table 1:** Overview of the different treatment regimens applied.

Drug/Antigen	Administration route	Start of administration	Amount per dose (doses distributed over 12 days in all cases)
α1-ECD_mt_	intranasal	D7	100 μg (12 doses)
	intravenous	D7	- 5 μg, 25 μg or 100 μg (12 doses) - 200 μg (6 doses) - 100 μg (6 doses) - 400 μg (3 doses) - 200 μg (3 doses)
	intravenous	D21	100 μg or 500 μg (12 doses)
	intravenous	D40	500 μg or 1000 μg (12 doses)
α1-ECD_m_	intravenous	D21	100 μg or 500 μg (12 doses)
	intravenous	D40	500 μg or 1000 μg (12 doses)
Pyridostigmine	intragastrical	D40	18.5 mg/Kg (12 doses)
Methylprednisolone	intraperitoneal	D40	1 mg (12 doses)

The rats were monitored once a week for the first 4 weeks after EAMG induction and daily thereafter. Body weight was recorded and clinical score was observed on a flat bench before and after exercise and graded based on the presence of the following symptoms: tremor, hunched posture, reduced strength/mobility and dropped head. Exercise consisted of repetitive grasping and pulling of a 350 g grid while being held by the base of the tail for 30 seconds (30). EAMG scores were evaluated as follows: 0: normal strength, no symptoms; 1: normal before exercise, symptoms observed after exercise due to fatigue; 2: symptoms present without exercise; 3: severe symptoms at rest, hind limb paralysis, no grip; 4: moribund (15). To minimize investigator bias, the animals were scored by two investigators, one of which was blinded to the treatment groups, and the average scores were used in the analyses.

### 2.4 Electromyography

The rats were anaesthetized with 2% isoflurane. To measure the compound muscle action potential (CMAP) the tibialis anterior muscle was examined. A grounding electrode was placed subcutaneously at the upper back; a stimulating electrode was inserted at the base of the tail to stimulate the sciatic nerve; a recording electrode was placed in the center of the tibialis muscle and a reference electrode more distally at the tendon. A set of 10 supramaximal stimuli at 3 Hz were delivered and the CMAP recorded. The average decrement for each muscle was calculated from at least three separate readings.

### 2.5 α1-ECD_mt_ Distribution Studies

^125^I -labeled α1-ECD_mt_ equivalent to 10^6^ cpm was mixed with unlabeled α1-ECD_mt_ to a total of 100 µg protein, which was injected intravenously to healthy or EAMG rats. Blood samples were collected from the tail artery at specific time points and organs were collected for analysis 6 hours after injection. Radioactivity was measured in a 1470 Wizard γ-counter. To calculate the organ distribution, the labelling attributed to the blood content of each organ was estimated and subtracted (31).

### 2.6 RIPA for Rat AChR and α1-ECD_mt_ Antibody Quantitation

α1-ECD_mt_ or α-bungarotoxin (Sigma-Aldrich, USA) were labeled with ^125^I using the chloramine T method. Following, the antibodies in test serum samples were quantified using RIPA. In brief, for the detection of α1-ECD_mt_ antibodies, ^125^I- α1-ECD_mt_ (50,000 cpm) was incubated for 2 h at 4°C with serial dilutions of the test serum (made in normal rat serum). The total volume of rat serum used was 2 μl. Then 10 μl of rabbit anti-rat serum were added and incubated overnight at 4°C. Finally, the samples were washed twice with PBS-T and the remaining radioactivity measured in a 1470 Wizard γ-counter. The dilutions showing a linear increase were used for the calculation of the antibody titers. For the detection of rat AChR antibodies, rat AChR was prepared from denervated rat muscle and labeled for 1 h at 4˚C with ^125^I- α-bungarotoxin (50,000 cpm) before incubation with the test serum serial dilutions. All the following steps were performed as described previously for the α1-ECD_mt_ antibodies.

To calculate the antibody titers in nM, the following formula was used:


$$\begin{array}{l} \text{Antibody titer}=(\text{cpm test serum sample}-\text{cpm of normal rat serum})/(\text{specific activity of labeled antigen x serum volume in μl}\times \text{dilution factor})\\ \text{where specific activity = cpm/fmol labeled antigen.} \end{array}$$

### 2.7 Statistical Analysis

**Figure 1:**
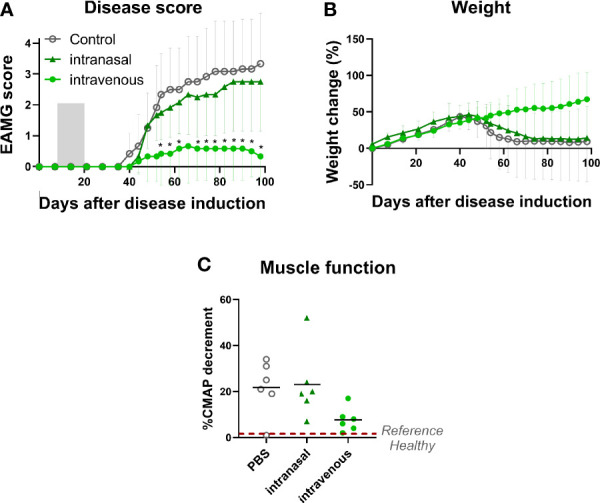
Intravenous administration of α1-ECD_mt_ results in a superior treatment effect compared to intranasal administration. EAMG rats received PBS *via* the intravenous route (N = 6, empty circles) or 100 μg α1-ECD_mt_ in PBS *via* the intranasal (N = 6, triangles) or the intravenous route (N = 6, filled circles) on twelve consecutive days, from day 7 to day 18 of the experiment. **(A)** Average EAMG score (± SD) for each group; the grey bar indicates the treatment period. **(B)** Average percentage of body weight change (± SD). **(C)** CMAP decrement: each symbol corresponds to one rat and the bar shows the mean value. * Compares intravenous α1-ECD_mt_ and control.

**Figure 2:**
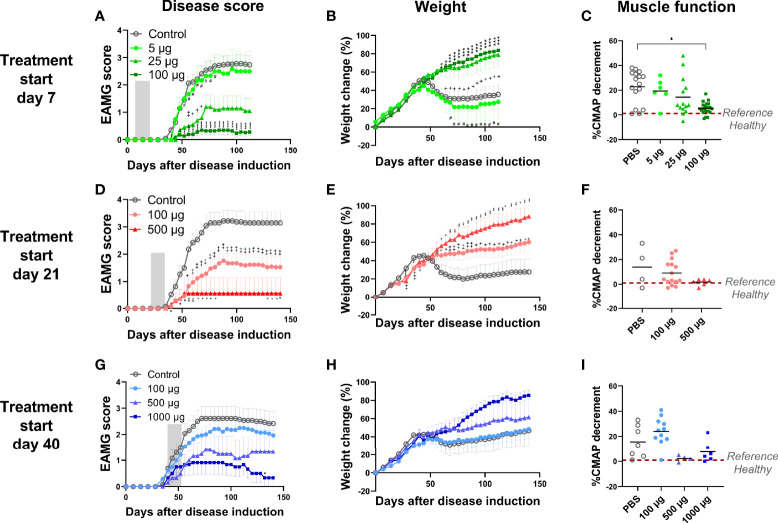
Intravenous administration of α1-ECD_mt_ abrogates EAMG development in a dose and time dependent manner. **(A-C)** The data are compiled from 3 independent experiments. Rats were treated starting on day 7 with 5 μg (N = 6, circles), 25 μg (N = 14, triangles), 100 μg α1-ECD_mt_ (N = 23, squares) or PBS (N = 23, open circles). **(D-F)** The data are compiled from 2 independent experiments. Rats were treated starting on day 21 with 100 μg (N = 21, circles), 500 μg α1-ECD_mt_ (N = 7, triangles) or PBS (N = 14, open circles). **(G-I)** The data are compiled from 2 independent experiments. Rats were treated starting on day 40 with 100 μg (N = 14, circles), 500 μg (N = 6, triangles), 1000 μg α1-ECD_mt_ (N = 6, squares) or PBS (N = 13, open circles). Grey bars, **(**in **A, D, G**) indicate the treatment period. The EAMG score (average ± SEM), percentage of body weight change (average ± SEM), and CMAP decrement were measured (for the later each symbol corresponds to one rat and the bar shows the mean value). In **(A-C)** # compares 5 µg vs 100 µg, + compares PBS vs 25 µg, and * compares PBS vs 100 µg. In **(D–F)** + compares PBS vs 100 µg and *compares PBS vs 500 µg. In **(G-I)** * compares PBS vs 1000 µg.

**Figure 3:**
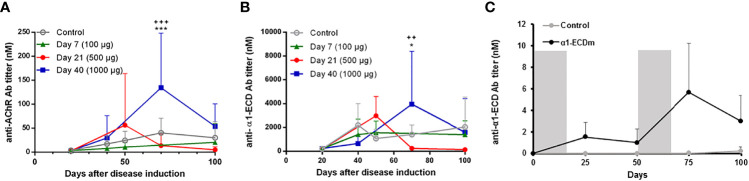
Serum levels of rat AChR and α1-ECD_mt_ antibodies following intravenous administration of α1-ECD_mt_. **(A)** Serum samples were collected at the indicated time points after disease induction from rats treated with 100 μg α1-ECD_mt_ on day 7 (N =, triangles), 500 μg on day 21 (N=7, circles), 1000 μg on day 40 (N = 6, squares), or PBS (N = 23, open circles), and were analyzed by RIPA for rat AChR antibodies. **(B)** The same serum samples as in **(A)** were assayed for α1-ECD_mt_ antibodies. **(C)** Healthy rats were injected with 500 μg α1-ECD_mt_ or PBS (N=10 for each group) during days 0 – 12 and days 50 – 62 as indicated by the gray bars, and serum samples collected at the indicated time points were analyzed for rat AChR and α1-ECD_mt_ antibodies (the lines show only α1-ECD_mt_ antibody titers, AChR antibodies were not detectable). In **(A, B)** * compares PBS vs Day 40, + compares Day 21 vs Day 40.

**Figure 4:**
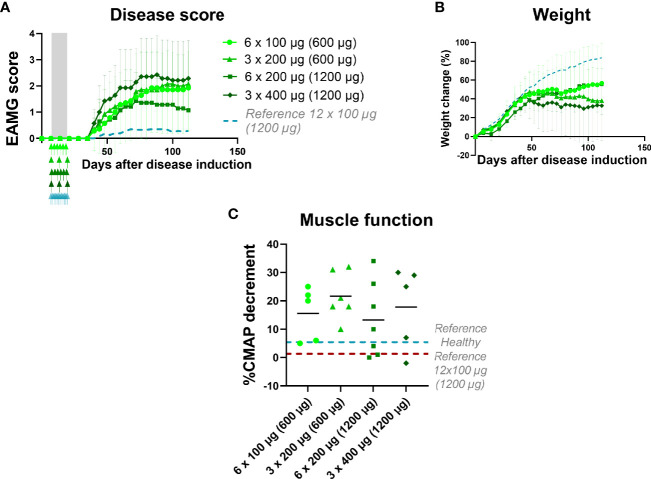
EAMG treatment efficacy of α1-ECD_mt_ depends on dosing frequency. EAMG rats received α1-ECD_mt_ at different doses and frequencies starting from day 7 of the experiment and over 12 days: 100 μg every other day (N=7, circles; 600 μg in total), 200 μg every other day (N=7, triangles; 1200 μg in total), 200 μg every fourth day (N=7, squares; 600 μg in total), or 400 μg every fourth day (N=7, rhombus; 1200 μg in total). **(A)** Average EAMG scores (± SD); the grey bar indicates the treatment period and arrows the timing of each administration. A dotted line represents rats treated with 12 x 100 μg daily administrations for reference. **(B)** Average percentage of body weight change (± SD). **(C)** CMAP decrement: each symbol corresponds to one rat and the bar shows the mean value.

**Figure 5:**
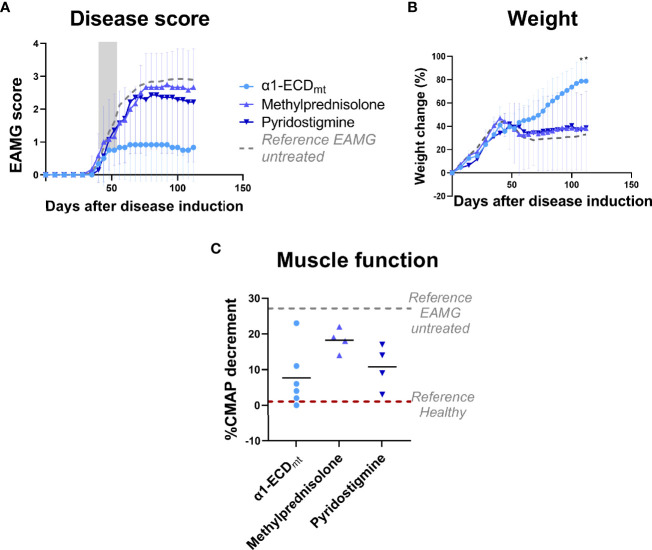
EAMG treatment efficacy of α1-ECD_mt_ is superior to that of two different active treatments for MG. EAMG rats were treated with either 5 mg/Kg methylprednisolone administered intraperitoneally (N = 7), 18,5 mg/Kg pyridostigmine administered orally (N = 6), or 1000 μg α1-ECD_mt_ administered intravenously (N = 6) on twelve consecutive days, starting at day 40 after disease induction. **(A)** Average EAMG scores (± SD); the grey bar indicates the treatment period. Circles correspond to α1-ECD_mt_, triangles correspond to methylprednisolone and inverted triangles correspond to pyridostigmine. **(B)** Average percentage of body weight change (± SD). **(C)** CMAP decrement: each symbol corresponds to one rat and the bar shows the mean value. In **(C)** dotted lines represent untreated EAMG rats and healthy rats for reference. *Compares α1-ECD_mt_ and methylprednisolone.

Statistical analysis was performed using GraphPad Prism (GraphPad Software, La Jolla, CA). In **Figures 1**–**5** when comparing at least three groups, for EAMG scores and CMAP decrement the two-tailed nonparametric Kruskal Wallis test was used. All other comparisons were made using two-tailed ANOVA. In **Figure 7** when comparing only two groups, for EAMG scores and CMAP decrement the two-tailed nonparametric Mann Whitney test was used. All other comparisons were made using unpaired t-test. For all figures: ****p < 0.0001, ***p < 0.001, **p < 0.01, *p < 0.05.

## 3 Results

### 3.1 Administration of α1-ECD_mt_ *via* the Intravenous Route Greatly Enhances Treatment Efficacy

We used a mutated and tagged version of the α1-ECD with vastly improved solubility (α1-ECD_mt_), near native conformation and practically identical binding to autoantibodies from MG sera, compared to the *wt* protein (26, 32) that could be used as an antigen-specific tolerogen. Initially, we investigated the potential of therapeutic intravenous antigen administration, using our robust rat model of EAMG (**Supplementary Figure 1**).

To this end, we treated EAMG rats daily on 12 consecutive days from day 7 after disease induction with 100 μg of α1-ECD_mt_, administered by intravenous injection or intranasal droplets. We observed a highly significant improvement in treatment effect after intravenous administration compared to intranasal administration (**Figure 1**). In fact, most of the intravenously α1-ECD_mt_ -treated rats remained asymptomatic (4/6 had an EAMG score of 0), while 5/6 of the intravenously PBS-treated rats presented with very severe disease. Similarly, the body weight and CMAP measurements of the intravenously treated animals were improved (**Figures 1B, C**).

### 3.2 α1-ECD_mt_ Abrogates EAMG Development in a Dose and Time Dependent Manner

To investigate if α1-ECD_mt_ demonstrated a dose-response relationship we injected the drug candidate at different doses ranging from 5 µg to 100 µg, starting treatment on day 7 as previously. We observed a strong correlation between the dose of α1-ECD_mt_ and treatment outcome, with higher doses having increased therapeutic efficacy. Indeed, rats treated with the 5 μg dose showed minimal improvement as their EAMG score reached 2.58 (± 0.72), while those that received 25 μg and 100 μg had an average maximum score of 1.2 (± 0.45) and 0.35 (± 0.16) respectively (**Figure 2A**). These findings were corroborated by the changes seen in body weight and CMAP decrement of the rats (**Figures 2B, C**).

Since the ultimate goal of antigen-specific tolerization is to treat ongoing autoimmune disease, we further studied the therapeutic effect of treatment with α1-ECD_mt_ following intravenous administration initiated at later timepoints after EAMG induction, specifically day 21 or 40. At these timepoints, EAMG rats display progressive disease both at the molecular and the clinical level, at different severities (subclinical to first symptom appearance). We found that treatment starting at these later time points was still effective but required higher drug doses to achieve comparable treatment effects to those obtained at earlier time points. As seen in **Figure 2D**, administration of 100 μg doses per day starting on day 21, resulted in an improvement of the EAMG score compared to mock-treated rats, but less than when treatment was initiated on day 7. However, increasing the daily dosing to 500 μg α1-ECD_mt_ initiated at day 21, had a more robust effect. Similarly, when treatment was initiated on day 40, the 100 μg dose group was less effective and not different from the PBS controls, while at higher doses of 500 μg or 1000 μg a profound improvement was achieved (**Figure 2G**). As previously, further evidence to these findings was given by measurements of the changes in body weight and CMAP decrement (**Figures 2E, F, H, I**). Following, we measured the rat AChR and α1-ECD_mt_ antibody titers of the highest dosed animals at different time points. We saw that treatment resulted in reduction of the AChR antibodies compared to PBS treated rats when administered on day 7, while treatment on days 21 or 40 post induction resulted in an antibody increase (**Figure 3A**). Similarly, α1-ECD_mt_ antibodies showed an increase in rats treated at the later time points (**Figure 3B**). However, there was no correlation between disease score and levels of total rat AChR antibodies or α1-ECD_mt_ antibodies upon treatment (**Supplementary Figure 2**), suggesting specific antibody characteristics influence their role. Furthermore, healthy 6 week-old rats injected with 500 μg protein *i.v.* in two sessions (12 daily injections each session) developed low levels of α1-ECD_mt_ antibodies, did not have detectable rat AChR antibodies and did not develop EAMG symptoms (**Figure 3C**). Notably, at the highest dose investigated, with a short-term dosing regimen, applied at a time point of established disease, α1-ECD_mt_ could ameliorate disease symptoms.

### 3.3 EAMG Treatment Efficacy of α1-ECD_mt_ Depends on Dosing Frequency

We next sought to determine the effect of dosing schedule and the impact on treatment efficacy in relation to dosing intervals. To this end, we compared the therapeutic effect of α1-ECD_mt_ injected intravenously in EAMG rats, every other day or every four days, over a twelve-day-period. The total amount of α1-ECD_mt_ administered over the entire treatment period was either 1200μg (in the 6 x 200 μg and 3 x 400 μg groups) or 600 μg (in the 6 x 100 μg and 3 x 200 μg groups). The results obtained indicate that treatment effect of frequent administrations of lower doses of α1-ECD_mt_ is superior to less frequent administrations of higher doses (**Figure 4**). Indeed, 6 administrations of 200 μg was more effective compared to 3 administrations of 400 μg. Similarly, administration of 6 x 100 μg was somewhat better than 3 x 200 μg, or even 3 x 400 μg, although the difference was not as profound, possibly due to the lower total antigen dose administered (600 μg), which yielded an overall moderate therapeutic effect. Taken together, the therapeutic efficacy of α-ECD_mt_ in the EAMG model is enhanced by continuous daily dosing of drug over 12 days compared to fewer administrations, of higher drug doses, over the same period.

### 3.4 EAMG Treatment Efficacy of α1-ECD_mt_ Is Superior to that of two Different Active Controls

To evaluate whether α1-ECD_mt_ treatment could confer significant benefit in the EAMG model over current mainstay treatments for patients with MG, we compared it to a cholinesterase inhibitor (pyridostigmine) and a corticosteroid (methylprednisolone) commonly used in clinical practice. As seen in previous experiments, treatment with α1-ECD_mt_ starting on day 40 resulted in remission of EAMG symptoms (**Figure 5**). In contrast, treatment with the cholinesterase inhibitor or the corticosteroid over the same period had reduced efficacy compared to the test antigen.

### 3.5 α1-ECD_mt_ has a Short Plasma Half-Life and Is Distributed to the Liver, Kidneys, and Spleen Upon Intravenous Administration

**Figure 6:**
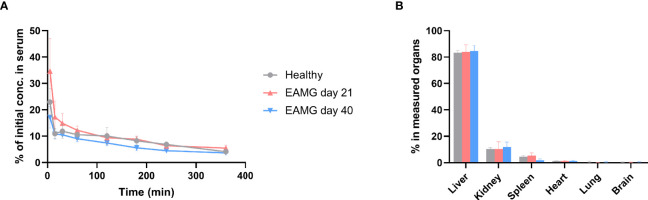
α1-ECD_mt_ has a short plasma half-life and is distributed to the liver, kidneys, and spleen upon intravenous administration. 100 µg ^125^I-labelled α1-ECD_mt_ (10^6^ cpm) were injected intravenously to healthy rats or EAMG rats on day 21 or day 40 after disease induction. **(A)** Blood was collected at various timepoints after intravenous injection and the residual ^125^I-labelled α1-ECD_mt_ was measured. 100% corresponds to the 10^6^ cpm originally injected. (N = 12 for each group) **(B)** The rats were sacrificed six hours after injection, selected organs were collected and measured. The value for each organ corresponds to recovered ^125^I-labelled α1-ECD_mt_ minus the value contributed by residual ^125^I-labelled α1-ECD_mt_ in the organ’s estimated blood content. (N = 12 for healthy and day 21 groups and N = 10 for day 40 group).

Upon intravenous administration of α1-ECD_mt_ to either healthy or EAMG rats, on day 21 or day 40 after disease induction, plasma levels of α1-ECD_mt_ followed a biphasic curve, with a steep distribution phase followed by a shallow elimination phase (**Figure 6A**). Importantly, there was no significant difference in distribution and elimination of α1-ECD_mt_ after administration to either healthy or EAMG rats, indicating that the presence of α1-ECD_mt_ antibodies in the EAMG rats did not affect the drug pharmacokinetics.

We studied the organ distribution of α1-ECD_mt_ 360 _min_ after intravenous injection into healthy or EAMG rats on day 21 or day 40 after disease induction. Uptake, calculated as the percentage of measured protein in all organs examined, was predominant in the liver, and to a lesser extent in the kidneys and spleen, while uptake into heart, lung, and brain was negligible (**Figure 6B**). As for plasma clearance, organ distribution was not affected by the stage of disease development, nor by the absence or presence of α1-ECD_mt_ antibodies.

### 3.6 α1-ECD_mt_ and α1-ECD_m_ Display Comparable Treatment Efficacies

**Figure 7:**
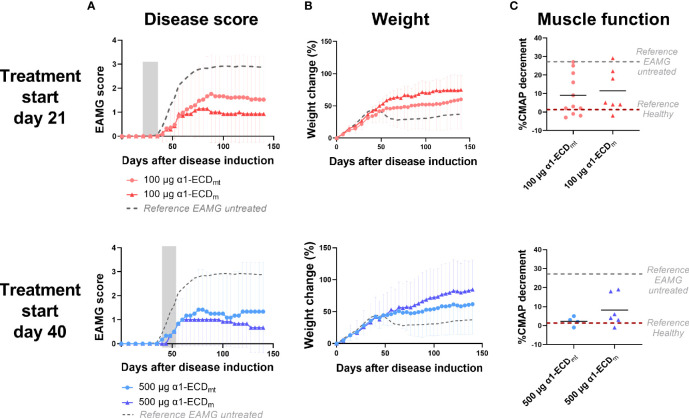
α1-ECD_mt_ and α1-ECD_m_ display comparable treatment efficacies. EAMG rats received α1-ECD_mt_ or α1-ECD_m_ by intravenous injection on twelve consecutive days from day 21 (100 μg; N = 14 for ECD_mt_ and N = 7 for α1-ECD_m_) or day 40 (500 μg; N = 6 for each group) of the experiment. **(A)** Average EAMG scores (± SD); the grey bar indicates the treatment period. Circles correspond to α1-ECD_mt_ and triangles correspond to α1-ECD_m_, while the dotted line represents untreated rats for reference. **(B)** Average percentage of body weight change (± SD). **(C)** CMAP decrement: each symbol corresponds to one rat and the bar shows the mean value. In **(C)** dotted lines represent untreated EAMG rats and healthy rats for reference.

In view of developing an antigen-specific therapy for MG suitable for use in the clinic, we re-engineered α1-ECD_mt_ by removal of the N- and C-terminal protein purification tags, and thus created α1-ECD_m_. These tags are extremely useful as research tools, enabling easy and efficient purification by affinity chromatography, but are inappropriate as parts of human protein therapeutics by posing an increased immunogenicity risk. Moreover, whereas α1-ECD_mt_ was produced in the yeast *P. pastoris*, we elected to produce α1-ECD_m_ in *E. coli* for ease of manufacturing and scale-up purposes. In experiments comparing intravenous treatment of EAMG rats with α1-ECD_m_ or α1-ECD_mt_, starting on day 21 or day 40 after disease induction with 100 μg or 500 μg doses respectively, we found comparable treatment efficacies of the two proteins (**Figure 7**). Taken together, these results obtained in the rat EAMG model point to α1-ECD_m_ as an antigen-specific therapeutic for translation to the clinic as a promising drug candidate for the treatment of patients with MG.

## 4 Discussion

Immunotherapies commonly used to treat MG are unspecific and associated with serious long-term side effects. To mitigate this, some immunotherapies currently in development are directed against pathological mechanisms more specific to MG (33, 34). These therapies, such as complement C5 inhibitors and neonatal Fc receptor inhibitors, may be associated with fewer side effects, but they will not reinstate tolerance and are, thus, not long-lasting or curative. Antigen-specific immunotherapies aiming to restore tolerance to the autoantigen under attack by specifically targeting only the part of the immune system that has gone awry while leaving the rest intact are, therefore, considered the holy-grail for treatment of autoimmune diseases.

Antigen-specific tolerization approaches based on the α1-ECD have been studied earlier, using the oral (35, 36) or intranasal (18, 37) but not the intravenous route of administration. Delivery *via* the intravenous route could exploit a natural noninflammatory path, readily perfusing several organs with resident immune cells which have developed unique mechanisms for induction and maintenance of tolerance.

Comparison of intravenous and intranasal administration in our animal model showed that the former was much more potent in treating EAMG rats. The low efficacy of intranasal treatment observed compared to published data from other groups, could be due to the relatively high severity of disease induced in our model, which, if left untreated, results in >95% symptomatic animals and high average clinical scores. The effect of intravenous treatment was clearly dose dependent, similar to what is seen in studies on i.v. tolerance in the EAE animal model (38). This effect could be due to binding of the injected α1-ECD_mt_ to circulating antibodies. However, since previous studies on aphaeresis of autoantibodies have shown that the therapeutic effect only lasts a few days after treatment termination (25), while in this case the benefit was long-lasting, we suggest that at least the lasting effect is mostly due to an additional mechanism, and further studies are needed to elucidate the contribution of each mechanism in the overall effect. Although a high therapeutic effect at later time points during disease progression was more difficult to achieve, possibly due to accumulation of extensive damage at the neuromuscular junctions as well as establishment of memory cells, increased doses of administered antigen were capable of ameliorating disease symptoms. Importantly, administration of the antigen was always performed after disease induction, rather than before (preventive treatment) when antigen-specific cells and antibodies are not yet present. In this setting, a possible influence of CFA on i.v. administration has not been determined and should be addressed in future studies. A limitation of the current animal model and experimental set up is the use of the same antigen for disease induction and for treatment, as this does not reflect the heterogeneous immunological environment of MG patients and may boost the therapeutic efficacy in the animal model. Therefore, studies using rats immunized with all AChR subunit ECDs or with torpedo AChR will help elucidate the therapeutic efficacy of the α1-ECD_mt_ against more heterogeneous autoimmune responses.

To investigate the dose dependency and the requirement for repeated administrations, we examined the fate of α1-ECD_mt_ after intravenous administration. The pharmacokinetic properties of α1-ECD_mt_ with its very short plasma half-life could explain the need for larger doses and repeated administration as these measures may increase exposure of the protein to relevant tissues and cells involved. Pharmacokinetics of intravenously administered molecules is dictated by properties such as charge, size, shape, solubility and receptor interactions (39). Investigation of the protein’s biodistribution in major organs after injection revealed that most of what remains in the body is found in key organs, known to be involved in tolerance, namely the liver, kidneys, and spleen (40–47). Studies are underway, aiming to reveal the possible involvement of these organs and the specific cells involved in the mechanism of action to further our understanding of treatment following intravenous antigen administration and ultimately potentially allowing the design of therapeutics with enhanced targeting and longevity. Furthermore, the overall effect of α1-ECD i.v. injection on the normal function of the immune system needs to be investigated. We have conducted a preliminary non-GLP toxicological study, in which non-induced rats were dosed with 500 μg/rat/day of α1-ECD_m_ or PBS (3 per treatment). Serum IFN-γ, TNF-α, IL-2, IL-6, IL-10 and CRP were measured the day after the last injection, and no differences in any of the cytokine or CRP levels were observed between the two animal groups (data not shown). However, more extensive studies are required to fully address this.

Peptides have been successfully used for induction of mucosal tolerance in mouse EAMG as well as for i.v. tolerance in rat EAMG in complexes with class II MHC molecules (19, 20, 48). A hurdle in designing antigen-specific drug candidates is the polyclonal complexity of autoimmune diseases driven by distinct, diverse autoreactive immune cell repertoires of patients. Indeed, studies on peptide-induced tolerance in EAMG showed that individual AChR peptides failed to produce any therapeutic benefit, despite tolerization against those specific AChR epitopes, highlighting the difficulty in tolerance spreading over bystander epitopes (49, 50). The use of a protein autoantigen, presenting a majority of the auto-epitopes involved in the autoimmune disease in their native context, allows for patients’ individual antigen processing and presentation, thereby potentially reducing requirements associated with peptide-based approaches such as the need for dissection of immunodominant autoepitopes, production of personalized autoepitopes or patient stratification by HLA-type, and reliance on bystander effects to overcome patient heterogeneity.

Another reasoning for using T cell epitope peptides is to avoid administration of antigens bearing conformational epitopes that may be recognized by B cells. Studies in the rat EAMG model have shown that oral administration of an α1-ECD construct with more native conformation resulted in disease exacerbation, while treatment with a similar but less native fragment was able to suppress ongoing EAMG (36). However, in the case of intravenous administration, our results show that antigen conformation did not negatively affect the efficiency of treatment, since the *P. pastoris* protein used in most of the studies herein has a near native conformation (26), perhaps owing to differences in mechanism of action between the two administration routes.

While it is well understood that the molecular immunopathology in about 85% of patients with MG is due to the presence of circulating autoantibodies specifically targeting the AChR, the AChR antibody titer generally does not correlate with disease severity (51). Likewise, analysis of autoantibody levels in the rat EAMG model used here showed a weak correlation between disease score and the levels of total rat AChR antibodies measured in untreated animals and negligible correlation upon treatment (**Supplementary Figure 2**). In both cases, in agreement with previous findings (24), there was negligible correlation between clinical score and total α1-ECD antibodies (**Supplementary Figure 2**). The levels of total AChR and α1-ECD_mt_ antibodies were found increased in rats treated 21 or 40 days after disease induction. Similar results have been shown in some cases of oral administration, where improvement of clinical findings were accompanied by increased antibody titers (16). These results indicate that disease development and treatment effects seen in our studies are not linked to the entire heterogenic pool of antigen-specific antibodies but rather to a subset with particular pathogenic or tolerogenic qualities based on specificity, affinity, or isotype, as may also be the case in MG patients (51–54). Further experiments are required to dissect the qualities of the observed antibody responses after antigen injection in EAMG and healthy rats, which are crucial for understanding their role. This should be addressed by investigation of their AChR binding, antigenic modulation and complement activation *in vitro* as well as their *in vivo* potential to induce passive transfer EAMG, or AChR reduction and neuromuscular junction damage at a subclinical level. In addition, the potential immunogenicity of the administered antigen must be fully explored in healthy animals and *in silico* to ensure the safety of the approach. Additional studies are also needed to elucidate the underlying immunological mechanisms at play in the observed therapeutic effect, by analyzing relative frequencies, antigenic responses and cytokine profiles of regulatory B and T cells.

Protein glycosylation has been found to be important in regulating immune responses, and different glycans can lead to proinflammatory or immunosuppressive signals, although the mechanisms are far from being fully understood (55). Glycosylation of antigen used for therapy has been reported to play a role in i.v. tolerance induction in the case of EAE (56). In our model of i.v. tolerance, however, protein glycosylation did not appear to play a major role, since there was no difference in the therapeutic efficacy between the *P. pastoris* and the *E. coli* expressed proteins. It is, nonetheless, possible that the effect is polysaccharide-specific, and the comparatively shorter polysaccharides added by *P. pastoris* do not have the effect of longer mannans, such as those from *S. cerevisiae* (56).

A comparative study of the therapeutic efficacy of α1-ECD and pyridostigmine and methylprednisolone, two current standard of care therapeutics for MG, showed that the former had a superior effect. In fact, the clinical symptoms of rats treated with either of the two current drugs were almost identical with those of untreated animals. This highlights the qualitative difference of treatment with α1-ECD, as it had a therapeutic effect under conditions where current drugs had reduced efficacy. Furthermore, while most drugs currently used to treat MG are associated with serious long-term side effects, based on *in silico* immunotoxicity data α1-ECD_m_ shows no risk of increased immunogenicity in humans and a preliminary toxicology study in rats showed that α1-ECD_m_ was well-tolerated with no adverse clinical observations of note (data to be presented elsewhere). We, therefore, believe that our findings support the development of i.v. antigen administration as a viable therapeutic option for MG, and α1-ECD as a potent drug candidate.

In conclusion, our study describes an effective treatment of disease in the EAMG model by intravenous delivery of a recombinant soluble major MG autoantigen, α1-ECD_m_. If these results can be translated to human MG, α1-ECD_m_ represents a promising and efficient therapeutic approach for antigen-specific treatment. In the EAMG model α1-ECD_m_ shows a dose dependent capacity to induce remission after a short two-week treatment regimen. Furthermore, it provides an alternative route for clinical translation of antigen-based treatment of autoimmune diseases, as the makeup of the recombinant protein, comprising multiple auto-epitopes present in their native context, reduces the need for personalized tailoring of peptide-based treatments to overcome interindividual variability. Importantly, it is conceivable to use the same approach for several other autoimmune diseases in which the autoantigen is known, thus broadening the impact of our findings.

## Data Availability Statement

The raw data supporting the conclusions of this article will be made available by the authors, without undue reservation.

## Ethics Statement

The animal study was reviewed and approved by Protocols Evaluation Committee, Hellenic Pasteur Institute, Athens, Greece.

## Author Contributions

KL, MF-S, BL and CF designed the experiments and analyzed the data. J-OA and RC developed α1-ECD_m_. KL, VB and JG performed the experiments. KL and MF-S prepared the figures and figure legends. KL and CF wrote the manuscript. All authors reviewed, edited and approved the final manuscript

## Funding

This work was supported by funds from Toleranzia AB. and the Hellenic Pasteur Institute.

## Conflict of Interest

MF-S, J-OA, JG and CF are employees of Toleranzia AB. KL, RC and BL have received consultation or service fees from Toleranzia AB. MF-S, J-OA, BL and CF are shareholders in Toleranzia AB. KL has received research support from Toleranzia AB.

The remaining authors declare that the researchwas conducted in the absence of any commercial or financial relationships that could be construed as a potential conflict of interest.

The funder was involved in the study design, collection, analysis, interpretation of data, writing of this article and the decision to submit it for publication.

## Publisher’s Note

All claims expressed in this article are solely those of the authors and do not necessarily represent those of their affiliated organizations, or those of the publisher, the editors and the reviewers. Any product that may be evaluated in this article, or claim that may be made by its manufacturer, is not guaranteed or endorsed by the publisher.

## Abbreviations

MG, myasthenia gravis; AChR, nicotinic acetylcholine receptor; α1-ECD, extracellular domain of the α1 subunit of the human acetyl choline receptor; α1-ECD_m_, mutated form of the α1-ECD; α1-ECD_mt_, mutated and tagged form of the α1-ECD; EAMG, experimental autoimmune myasthenia gravis; APC, antigen-presenting cell; i.v. tolerance, tolerance induction *via* intravenous injection of soluble antigen; CMAP, compound muscle action potential; PBS, phosphate-buffered saline (pH 7,4); PBS-T, PBS containing 1% Tween.
