# Supplementary material for: A Recombinant Acetylcholine Receptor α1 Subunit Extracellular Domain Is a Promising New Drug Candidate for Treatment Of Myasthenia Gravis
Source: Front Immunol. 2022 Jun 3;13:809106. doi: 10.3389/fimmu.2022.809106 (PMC9204200; doi:10.3389/fimmu.2022.809106)
Supplement: Supplementary file 1 [file DataSheet_1.docx]

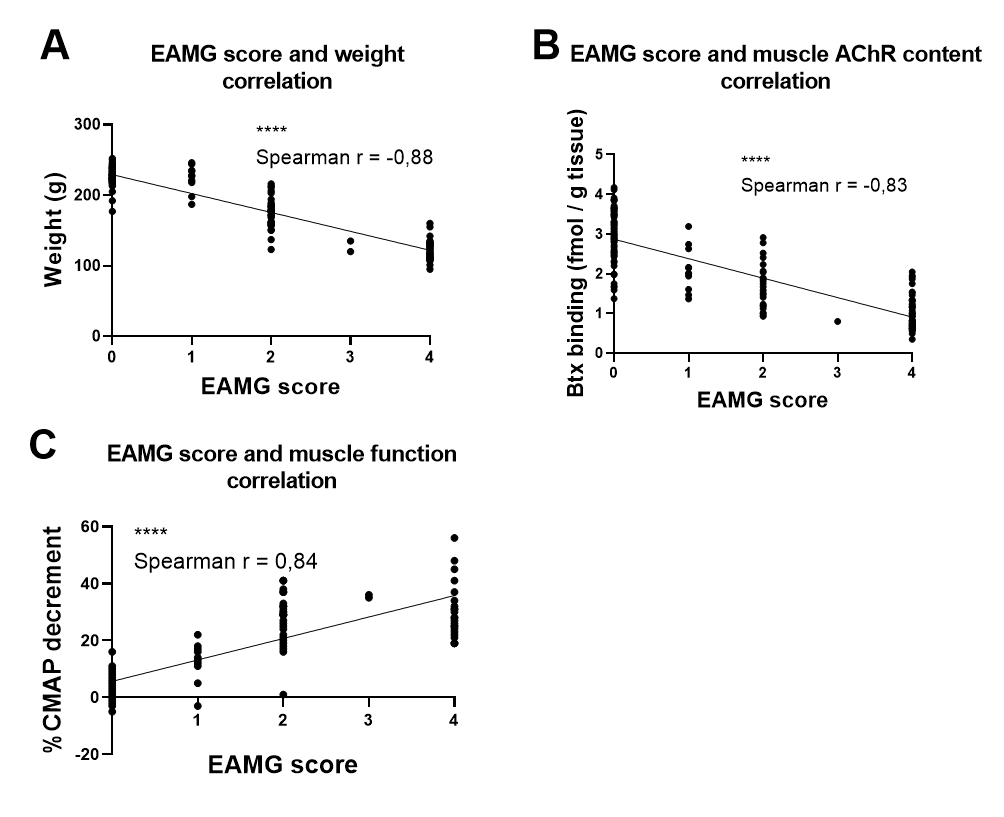


**Supplementary Figure 1.** **Robustness of the rat EAMG model.** Correlations between EAMG score and body weight on day 100 after disease induction **(A)**, muscle AChR content **(B)**, and muscle function presented as CMAP decrement **(C)**. The data are compiled from 132 EAMG rats over 7 separate experiments, where they were injected i.v. with PBS or different doses of α1-ECD_mt_ ranging from 5 to 1000 µg on twelve consecutive days starting on day 7, day 21, or day 40 after disease induction. Each symbol corresponds to one rat at a specific timepoint.
