# Supplementary material for: A Recombinant Acetylcholine Receptor α1 Subunit Extracellular Domain Is a Promising New Drug Candidate for Treatment Of Myasthenia Gravis
Source: Front Immunol. 2022 Jun 3;13:809106. doi: 10.3389/fimmu.2022.809106 (PMC9204200; doi:10.3389/fimmu.2022.809106)
Supplement: Supplementary file 2 [file DataSheet_2.docx]

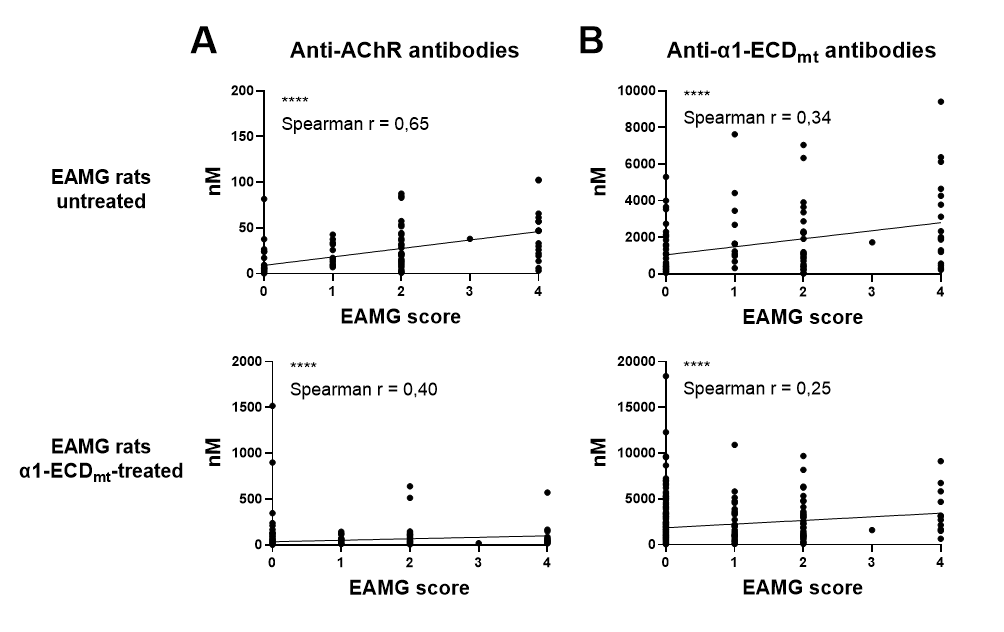


**Supplementary Figure 2.** **Correlation between disease score and antibody titers in the EAMG model.** EAMG rats received either PBS or different doses of α1-ECD_mt_ ranging from 5 to 1000 µg by intravenous injection on twelve consecutive days starting on day 7, day 21, or day 40 after disease induction. Correlation between EAMG score and anti-AChR antibodies **(A)** or anti-α1-ECD_mt_ antibodies **(B)** in untreated (N=42, upper panels) and treated rats (N=90, lower panels). Each symbol corresponds to one rat at a specific timepoint.
